# Evaluating participant experiences and tolerability with MR Linac imaging

**DOI:** 10.1016/j.tipsro.2025.100348

**Published:** 2025-10-03

**Authors:** Jayde Nartey, Helen A. McNair, Katie Biscombe, Sophie E. Alexander, Charlotte Cherry, Cynthia Eccles, Trina Herbert, Shaista Hafeez, Kelly Jones, Francesca Mason, Simeon Nill, Hosna Mohammad, Kian Morrison, Bethany Williams, Robert Huddart

**Affiliations:** a The Royal Marsden NHS Foundation Trust, London, the United Kingdom of Great Britain and Northern Ireland; b The Institute of Cancer Research, London, the United Kingdom of Great Britain and Northern Ireland; c The Christie NHS Foundation Trust, Department of Radiotherapy, Manchester, the United Kingdom of Great Britain and Northern Ireland; d Joint Department of Physics at the Royal Marsden and The Institute of Cancer Research, the United Kingdom of Great Britain and Northern Ireland; e Division of Cancer Sciences, The University of Manchester, Manchester, the United Kingdom of Great Britain and Northern Ireland

**Keywords:** Scan anxiety, MRI-guided radiotherapy, MR Linac, Tolerability

## Abstract

**Introduction:**

Magnetic Resonance Image Guided Radiotherapy (MRIgRT) integrates MRI with a linear accelerator to enable adaptive treatment delivery. While technical feasibility is well established, patient experience during MR Linac imaging, especially outside treatment sessions, remains underexplored. This study evaluates tolerability, pre-scan anxiety, coping ability, willingness for future scans, and scan-induced symptoms in patients and non-patient volunteers.

**Materials and Methods:**

Participants who successfully underwent MR Linac imaging between November 2017 and December 2023 completed a bespoke MR Linac Participant Experience Questionnaire, developed by the PRIMER study team and informed by MRI patient experience literature. The questionnaire assessed pre-scan anxiety, coping, willingness for future scans, and scan-related symptoms using Likert-scale responses. Descriptive analyses summarised responses by participant group and anatomical site.

**Results:**

In total, 447 participants (319 patients; 128 non-patient volunteers) completed MR Linac imaging and the questionnaire. Overall tolerability was high, with 65 % strongly disagreeing they felt anxious prior to scanning and most participants reporting good coping ability. Variability was observed across anatomical sites: Participants undergoing head and neck, brain, and oligometastatic bone scans reported higher pre-scan anxiety, with coping difficulties most frequently reported by the head and neck group. Non-patient volunteers reported more physiological symptoms (e.g., sweating, nausea, dizziness) than patients, whereas patients undergoing head and neck and bone oligometastases scans were more reluctant to repeat the procedure. Most patients perceived MR Linac imaging as easier or comparable to diagnostic MRI, though 20 % of brain cancer patients found it more difficult.

**Conclusion:**

MR Linac imaging is generally well tolerated, though specific subgroups, particularly those requiring immobilisation, report greater anxiety and discomfort. These findings highlight the need for tailored strategies to improve patient experience, supporting wider implementation of MRIgRT.

## Highlights

• Evaluated patient and non-patient experiences with MR Linac imaging across anatomical sites.
• Identified variation in anxiety, coping, and willingness for future MR Linac scans.
• Brain, head and neck, and bone metastases groups reported higher pre-scan anxiety.
• Suggest tailored strategies to improve tolerability and scan comfort..
• Findings support wider MR-guided radiotherapy use by improving patient experience.

## Introduction

Magnetic Resonance Image Guided Radiotherapy MRIgRT) is achieved using a hybrid system that combines MR imaging with the precise radiotherapy treatment capabilities of a linear accelerator [[1], [2], [3], [4]]. The MR Linac can utilise the superior imaging capabilities of MRI to aid in soft tissue enhancement [5]. MRIgRT allows for daily visualisation of changes in size, shape, and the position of the tumour and the surrounding organs at risk. One of its key advantages is its capacity for daily treatment adaptation. This adaptive capability enables real-time modifications to the treatment plan in response to anatomical changes observed during each session [6]. The ability to continuously monitor the tumour position during treatment to ensure the dose remains directed at the target area can improve patient outcomes by allowing for margin reduction and minimising damage to healthy tissue. [[7], [8], [9], [10]].

Patient experience and acceptability are key when assessing the feasibility of advanced imaging and treatment techniques such as MRIgRT; positive experiences can enhance compliance and cooperation during procedures. Creating a stress-free and comfortable experience will enable the patient to tolerate scanning, thereby enhancing the effectiveness of ongoing treatment. Factors such as pre-scan anxiety, scan duration, physical comfort, noise levels and overall environment can influence how well MRI procedures are tolerated [11]. Addressing these factors through tailored interventions, can directly impact patient experience and imaging outcomes [[12], [13], [14], [15], [16], [17]].

While numerous studies have explored patient experiences with MRI procedures in general [[11], [12], [13], [14], [15], [16], [17]], research specifically examining patient reported experiences and tolerability during imaging on MR Linac systems remains comparatively limited. Recent research on MR-guided radiotherapy has identified several factors influencing comfort and treatment compliance. For example, in one study although over 80 % of patients treated on a 1.5 T MR Linac found it moderately or very easy to maintain the treatment position, approximately 35 % reported wanting to come out of the scanner and 60 % forced themselves to tolerate the situation [18]. Similarly, in a prospective single-institution study of 204 patients, among all the questions asked, the one most frequently associated with participants reporting high levels of anxiety: “Self-control was required when going through the examination” [19]. Another study reported that while many patients appreciated the precision offered by MRIgRT, discomfort and anxiety were frequently linked to longer scan durations and use of immobilisation devices [20]. In a separate investigation of healthy volunteers in a fully enclosed MR-guided rotation system, compliance was generally high, but several individuals reported challenges related to physical discomfort and scan tolerability.

However, despite these important contributions, few studies have specifically focused on tolerability during standalone MR Linac imaging sessions outside the context of treatment delivery. Furthermore, existing research has rarely compared patient experiences directly with those of non-patient volunteers, nor systematically evaluated symptoms across different anatomical sites.

This study forms part of the PRIMER feasibility study (NCT02973828), a multi-stage protocol aimed at optimising MR Linac imaging workflows for radiotherapy. PRIMER was designed collaboratively by teams at The Royal Marsden, The Institute of Cancer Research (ICR), and The Christie NHS Foundation Trust. It aims to address gaps by providing detailed insights into pre-scan anxiety, coping ability during scanning, willingness for future scans, and scan-induced symptoms among both patients and non-patient volunteers undergoing MR Linac imaging as part of the PRIMER study. These insights can inform targeted strategies to improve the tolerability and patient experience of MR Linac imaging, thereby supporting broader clinical adoption of MRIgRT.

Standalone MR Linac imaging sessions as used in this study, differ from treatment sessions in that they do not involve radiation delivery or adaptive planning steps. As such, these sessions are generally shorter in duration and focus solely on acquiring imaging for research purposes, rather than for treatment positioning or verification. This may influence participant experience due to reduced time on the treatment couch and absence of radiation related anxiety.

## Methods and materials

The PRIMER study is structured into four stages (APENDIX B), aimed at enhancing tumour visualisation, reducing observer variability, refining the imaging process, and improving the patient experience to optimise imaging protocols and treatment workflows for MRIgRT on the MR Linac overall. Patients and non-patient volunteers were included in all stages, and imaging protocols were evaluated across different anatomical sites.

### Participant recruitment

Participants were recruited to research scans on the 1.5 Unity MR Linac Linac (Elekta AB, Stockholm Sweden) based on specific safety and suitability criteria. Non-patient volunteers were required to complete and pass an MRI safety screening, have no significant medical conditions, be over 18 years old and be able to provide informed consent. Patients were required to complete and pass an MRI safety screening, to be under the care of a Clinical Oncologist at RMH or CNHSF and be scheduled for radiotherapy with a confirmed cancer diagnosis; they were also required to have an ECOG performance status of 0–2. Participants were excluded if they failed the MRI safety screening due to certain medical implants or conditions that increase MRI-related risks.

Non-patient volunteers were recruited internally at ICR, RMH or CH; eligible individuals were approached and invited to participate. Eligible patients were identified by the radiotherapy teams at both hospitals and informed about the study.

Participant numbers reported in this study reflect individuals who successfully underwent MR Linac imaging and completed the participant experience questionnaire. Prior to recruitment, all potential participants were screened for MRI safety contraindications, including metallic implants, pacemakers, or other conditions incompatible with MRI. Individuals identified with contraindications at this stage were not approached for consent, did not receive a Patient Information Sheet (PIS), and were excluded from the eligible pool.

This analysis includes all participants scanned between November 2017 and December 2023 as part of the PRIMER study. A total of 498 eligible patients were invited to participate and consented, of whom 21 withdrew before scanning due to reasons such as illness. Only participants who completed both the MR Linac scan and the participant experience questionnaire were included in this analysis.

### Patient experience questionnaire

Participant experiences were assessed using an adapted MR Linac Participant Experience Questionnaire (Appendix A). The questionnaire used in this study was developed by the PRIMER study team. Its content was informed by common themes identified in prior MRI patient experience literature [22] and aligned closely with the domains explored in later validated MR Linac treatment experience tools [18]. The questionnaire was designed to evaluate key aspects of tolerability and experience relevant to MR Linac imaging, covering the following:

• Pre-scan anxiety (e.g. “I was anxious about my scan before I had it done.”).
• Coping during the scan (e.g. “I had difficulties coping with the scan.”).
• Willingness for future MR Linac scans (e.g. “I would not be worried about having more of these scans.”).
• Comparison to prior diagnostic MRI scans (for participants with previous MRI experience).
• Physical symptoms experienced during the scan (e.g. discomfort, tingling, sweating, nausea, dizziness).

Responses were recorded using a 5-point Likert scale ranging from “Strongly disagree” to “Strongly agree.” Participants were also monitored during and immediately after scans, with follow-up contact within 24 h to check for any adverse events. For this analysis, all completed questionnaires were included, acknowledging that some participants contributed multiple responses. Data was collected from November 2017 to December 2023.

### Data analysis

All data analyses were descriptive. Results were summarised using frequencies and percentages for categorical responses. No statistical hypothesis testing was performed, as the primary objective was to describe participant experiences rather than to compare groups statistically.

### Ethical considerations

Participants received a PIS detailing the study's information, highlighting the purpose, procedure, potential risks, and benefits. Informed consent was obtained prior to the scanning procedure and maintained throughout. Considerations were made for vulnerable populations, such as minors and individuals with impaired decision-making capacity. In the case of paediatric participants, consent was obtained from their parent or legal guardian and the child, if appropriate.

The Stanmore Research Ethics Committee reviewed and approved the study protocol. Continuous safety monitoring was conducted throughout the study, and procedures were in place for immediate intervention if necessary.

## Results

A total of 447 participants, including 319 Patients **(**Table 1**)** and 128 Non-Patient Volunteers **(**Table 2**),** completed detailed questionnaires following their scan sessions, with a total of 711 patient and 185 non-patient volunteer questionnaires analysed.

**Table 1:** Patients characteristics by site.

Anatomical site	Number of Participants (%)
Bladder	27 (8.5 %)
Brain	3 (0.9 %)
Breast	10 (3.1 %)
Gynae	30 (9.4 %)
Hepato-pancreato-biliary (HPB)	48 (15.0 %)
Intra-abdominal paediatric (IAP)	7 (2.2 %)
Lung	43 (13.5 %)
Oligometastases (bone)	8 (2.5 %)
Oligometastases (soft tissue)	9 (2.8 %)
Oropharynx/larynx/hypopharynx	58 (18.2 %)
Prostate	38 (11.9 %)
Rectum	38 (11.9 %)
Total	319

**Table 2:** Non-Patient Volunteer characteristics by site.

Anatomical region	Number of Participants (%)
Abdomen	27 (21.1 %)
Head and Neck	21 (16.4 %)
Pelvis	32 (25.0 %)
Thorax	48 (37.5 %)
Total	128

**Fig. 1:**
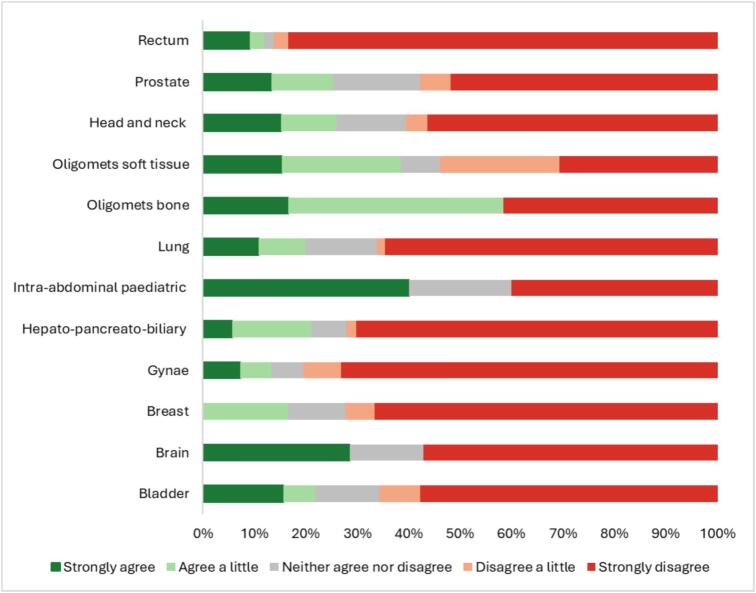
Patients Pre-Scan Anxiety by Anatomical Tumour Site – ‘I was anxious about my scan before I had it done’.

Pre-scan anxiety differed across anatomical sites. Patients having IAP scans exhibited the highest level of anxiety, with 40 % strongly agreeing to feeling anxious. Patients diagnosed with rectal cancer and undergoing rectal scans experienced the lowest levels of anxiety, with 83 % of participants strongly disagreeing to any levels of anxiety (Fig. 1). A substantial proportion of brain cancer patients reported pre-scan anxiety. However, most also reported good coping ability and willingness to undergo future scans.

**Fig. 2:**
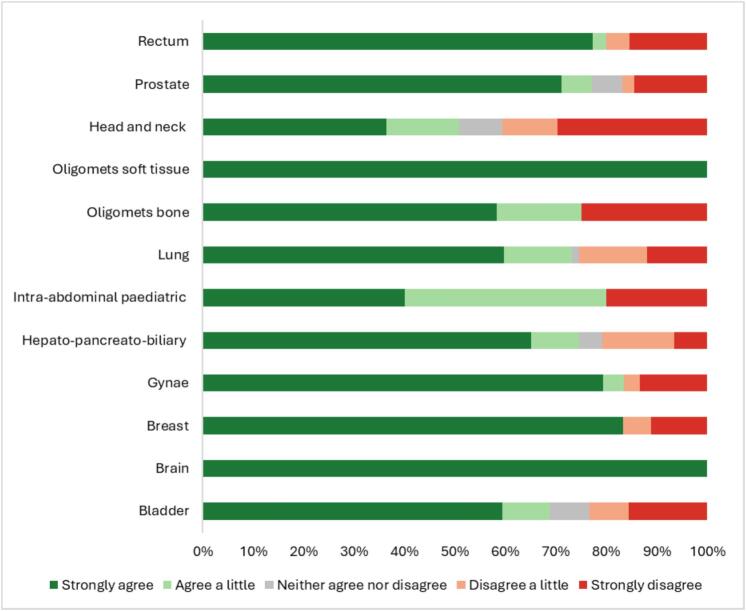
Patients Ability to Cope During Scanning by Anatomical Tumour Site imaged – ‘I had no difficulties coping with the scan’.

Patients diagnosed with brain cancer demonstrated the highest coping ability, with 100 % strongly agreeing that they did not experience any difficulties. Those undergoing head and neck scans reported the worst coping abilities, with 30 % strongly disagreeing **(**Fig. 2**)**.

**Fig. 3:**
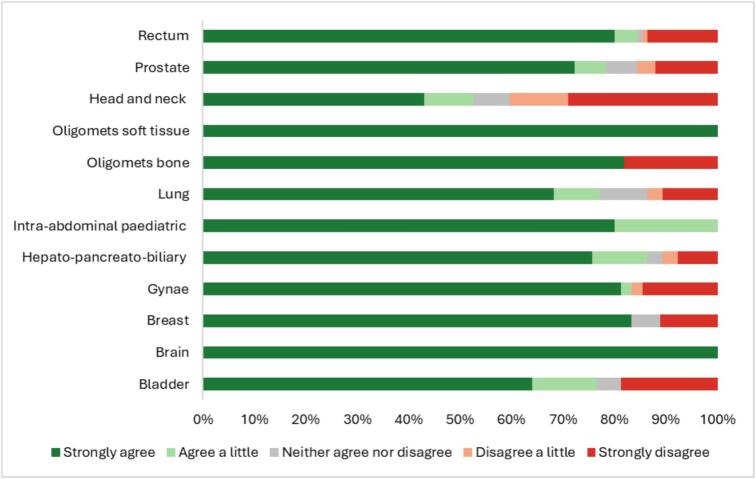
Patients Willingness to Undergo Future MR Linac Scans by Anatomical Tumour Site – ‘I would not be worried about having more of these scans’.

The analysis of patients willingness to undergo future MR Linac scans revealed variability between anatomical sites. Patients diagnosed with brain tumours and those with soft tissue oligometatases showed the lowest levels of reluctance with 100 % of these participants being open to future scans. In contrast, patient diagnosed with head and neck cancer reported significant hesitancy with 29 % strongly disagreeing to undergoing future scans **(**Fig. 3**)**.

**Fig. 4:**
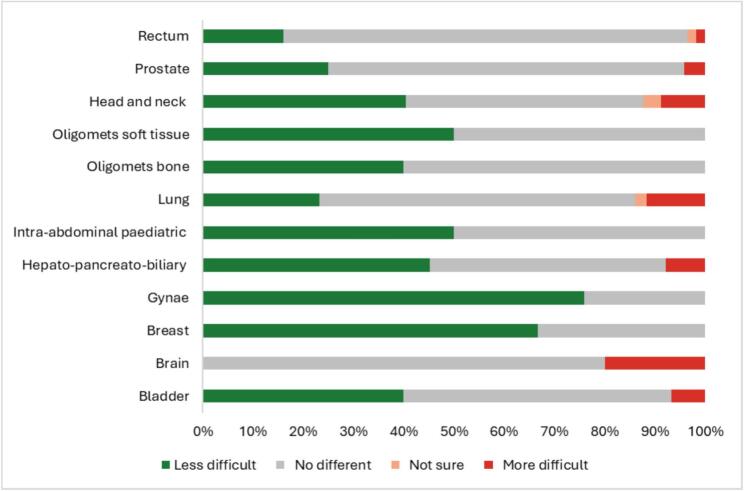
Patients Comparison of MR Linac To diagnostic MR by Anatomical Tumour site − ‘Compared to my diagnostic MRI, the MR Linac was…’.

When comparing the difficulty of MR Linac scans to previous diagnostic MRI scans, 76 % of gynaecological cancer patients found MR Linac scans less difficult. However, 20 % of brain cancer patients and 12 % of lung cancer patients found MR Linac scans to be more difficult. In contrast, 80 % of rectal cancer patients found no difference between scans (Fig. 4).

**Fig. 5:**
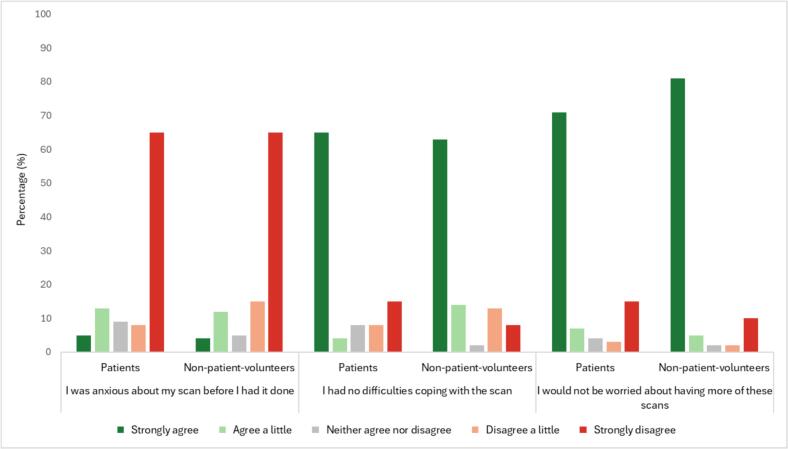
Participant Experience Responses – Patients vs Non-Patient Volunteers.

The analysis of participant experiences responses for both patients and non-patient volunteers revealed that both groups reported low levels of pre-scan anxiety. Specifically, 65 % of both patients and non-patient volunteers strongly disagreed with feeling anxious before their scan, although non-patient volunteers showed slightly higher levels of mild anxiety. Patient volunteers were slightly more likely to “agree a little” that they were anxious (13 % vs 12 %). The ability to cope during scans was similar, with slightly more non-patient volunteers reporting difficulties than patient volunteers. When comparing the willingness to undergo future scans, non-patient volunteers showed a higher willingness to undergo future scans than patients, with 81 % (150/185 total responses) strongly agreeing compared to 71 % (495/702 total responses) of patients **(**Fig. 5**)**.

**Fig. 6:**
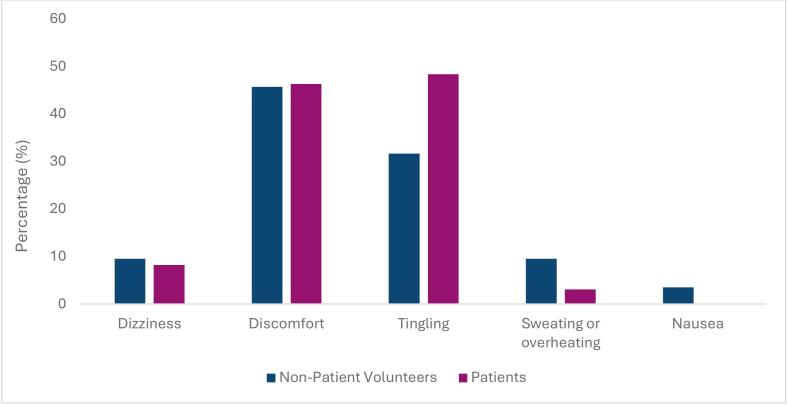
Physical Symptoms Experienced by Patients and Non– Patient Volunteers.

Discomfort and tingling were the most frequently reported symptoms experienced by all participants during MR Linac scans (Fig. 6). Non-patient volunteers appeared to experience more sweating, dizziness, and nausea compared to patients, with none of the patient group reporting nausea. 46 % of participants reported experiencing some level of discomfort and a tingling sensation, which was more frequent among non– patient volunteers (48 %) than patients (32 %).

## Discussion

This study provides one of the first detailed evaluations of participant experiences during MR Linac imaging outside treatment sessions, including both patients and non-patient volunteers. Overall, tolerability was high, with most participants reporting low levels of pre-scan anxiety, good coping ability, and willingness to undergo future scans. However, variation was observed across anatomical sites, with head and neck, brain, and bone oligometastatic patients reporting higher levels of pre-scan anxiety; coping difficulties were most common among those undergoing head and neck scans.

Participants with head and neck and brain tumours exhibited greater pre-scan anxiety and coping challenges, consistent with previous findings linking immobilisation devices and prolonged scan times to distress [[18], [19], [20],^23^,^24^]. Interestingly, brain cancer patients in this study reported strong coping ability despite initial anxiety, suggesting that effective in scan support may mitigate apprehension. These observations align with prior research showing that anxiety can often be managed through reassurance and tailored support [11,18,20]. This suggests that initial pre-scan anxiety may not necessarily translate into poor scan tolerance for this group, highlighting the potential benefit of reassurance and supportive measures prior to scanning. It is worth noting that brain cancer patients did not receive additional preparatory support beyond standard MR Linac imaging information, suggesting that standard measures may be sufficient for helping patients cope despite pre-scan anxiety.

This aligns with prior work by Barnes et al., who reported higher anxiety in head and neck patients undergoing MR Linac treatment sessions [18]. Moreira et al. and de Mol van Otterloo et al. also noted discomfort related to immobilisation and scan duration [19,20]. Beyer et al. highlighted physical challenges and symptoms such as dizziness and discomfort in MRI guided systems [21]. Unlike these studies, which focused on treatment contexts, our study examined standalone imaging sessions, highlighting experiences in the absence of therapeutic interventions and radiation delivery. This design allows us to isolate factors related to the imaging process itself.

Non-patient volunteers reported more physiological symptoms, such as sweating, nausea, and dizziness, compared to patients, possibly reflecting unfamiliarity with medical imaging environments. Nausea was exclusively reported by non-patient volunteers. The most common scan induced symptoms were tingling and discomfort, which are typically associated with MRI scanning [25]. The low severity of peripheral nerve stimulation (PNS) reported may be attributed to scans being performed in normal operating mode in accordance with MHRA guidance, which allows for mild PNS but prevents levels considered harmful [26].

Participants with bone oligometastates reported discomfort, possibly related to tumour-associated pain [27,28]. However, the small size of this subgroup limits the generalisability of findings and warrants cautious interpretation. Similarly, participants undergoing IAP scans, primarily adolescents and young adults, reported the highest pre-scan anxiety. Contributing factors may include unfamiliar environments, reduced prior imaging exposure, and communication barriers [29]. These results reinforce the need for age-appropriate preparation strategies, including child-friendly communication, visual aids, and environmental adaptations [30,31].

Willingness to undergo future MR Linac scans varied by anatomical site. Patients who underwent head and neck, bladder, and lung scans expressed greater reluctance, with 43 % of head and neck patients indicating a positive attitude toward repeat scans compared to 29 % who were reluctant. Previous research suggests that discomfort and anxiety during initial scans can reduce willingness for future imaging [[32], [33], [34]]. Addressing these concerns through pre-scan education, frequent reassurance during scans, and improved patient support may increase the likelihood of compliance for subsequent imaging [32,34,35,36].

When comparing MR Linac imaging to diagnostic MRI, most patients (93 %) found MR Linac scans to be easier or comparable, particularly for breast and gynaecological sites. However, for certain anatomical regions such as lung and HPB, patients perceived MR Linac imaging as more challenging, reporting increased difficulty compared to diagnostic MRI [37,38. Notably, 20 % of brain cancer patients also found MR Linac imaging more difficult, potentially due to longer scan times, immobilisation requirements, or neurological sensitivity in this group. Refinements to scanning protocols, such as optimising positioning or reducing scan time where feasible, may help mitigate these challenges [37,38].

These findings contribute novel insights by highlighting variations in MR Linac imaging experience based on anatomical region and patient type. They suggest that discomfort and anxiety are not uniformly experienced, and that even high anxiety groups may respond well when supported appropriately. Non-patient volunteer data further underscore the influence of prior exposure and familiarity with imaging environments.

To improve MR Linac imaging experiences, personalised pre-scan preparation should be considered. Strategies may include relaxation techniques, audiovisual aids, opportunities to ask questions, and enhanced environmental comfort [35,36,[39], [40], [41], [42]]. For those experiencing severe pre-scan anxiety, mild anxiolytic medication can be considered, although careful selection is essential to minimise adverse effects [43]. Additional comfort measures (e.g. padding, ventilation adjustments) may be especially valuable in complex anatomical regions such as head and neck. For high anxiety groups like adolescents or those with prior discomfort, targeted interventions such as child friendly materials or counselling should be explored [20,39,40].

A limitation of this study is the use of a non-validated, study-specific questionnaire, which may affect the reliability and generalisability of findings. However, the tool was informed by established MRI patient experience literature [11,18,20]. A validated MR Linac patient-reported experience measure has since been developed by our team [18] and should be adopted in future research. Other validated tools used in the literature [19] also offer opportunities for benchmarking and improvement. Additionally, as some participants contributed responses from multiple scanning sessions, this may introduce bias, particularly if repeat responders had different expectations or levels of familiarity compared to first-time participants. This should be considered in interpreting findings.

## Conclusion

This study provides detailed insights into participant experiences during MR Linac imaging across multiple anatomical sites. While overall tolerability was high, differences emerged between patient and non-patient volunteers, and among anatomical sites. Notably, brain and head and neck scans were associated with higher pre-scan anxiety and, in some cases, greater perceived scan difficulty. Non-patient volunteers reported more physical symptoms such as dizziness and nausea than patients. These findings highlight the importance of considering anatomical site and participant background when planning MR Linac imaging sessions. Future studies should explore tailored interventions to improve comfort and reduce anxiety, particularly in groups identified as more vulnerable to discomfort or distress.

## Declaration of competing interest

The authors declare that they have no known competing financial interests or personal relationships that could have appeared to influence the work reported in this paper.

## Appendix A

PRIMER – Participant Experience Questionnaire .

## Appendix B

### Stages of the PRIMER Study

The PRIMER study is structured into four stages, each aimed at enhancing tumour visualisation, reducing observer variability, refining the imaging process, and improving the patient experience to optimise imaging protocols and treatment workflows for MRIgRT on the MR Linac overall.

• Stage A: The use of MR imaging to optimise protocols for visualising normal tissue.
• Stage B: Patients for specific tumour types; oligometastases (bone or soft tissue), prostate, rectum, bladder, gynaecological, brain, lung, head and neck region, chest wall, paediatric abdomino-thoracic and hepato-pancreato-biliary region; underwent MR imaging to refine tumour visualisation.
• Stage C: To review observer variability and develop the adaptive workflow on the MR Linac, patients were scanned to establish optimal imaging protocols.
• Stage D: Both patients and non-patient volunteers were imaged to continuously evaluate and improve imaging MRIgRT on the MR Linac.
